# An Uncommon Presentation of Guillain-Barré Syndrome in a Young Postpartum Woman

**DOI:** 10.7759/cureus.93081

**Published:** 2025-09-24

**Authors:** Waqas Ahmed, Mohammed Zayan Nizam, Salman Rafi, Amer Khawaja

**Affiliations:** 1 Respiratory Medicine, Lancashire Teaching Hospitals NHS Foundation Trust, Chorley, GBR; 2 Cardiology, Lancashire Teaching Hospitals NHS Foundation Trust, Chorley, GBR; 3 Acute Medicine, Lancashire Teaching Hospitals NHS Foundation Trust, Chorley, GBR

**Keywords:** acute demyelinating neuropathy, cranial nerve involvement, guillain-barré syndrome, ivig treatment, postpartum complications

## Abstract

Guillain-Barré syndrome (GBS) is a heterogeneous condition, with demyelinating and axonal variants, often triggered by infections, though many cases occur without a clear antecedent. The postpartum period is increasingly recognized as a risk window for autoimmune conditions due to immune rebound following delivery. While GBS during pregnancy is uncommon, susceptibility rises in the early postpartum phase, particularly within the first three months. This report describes the case of a young woman who developed GBS in the third month postpartum following a vaginal delivery complicated by manual removal of a retained placenta. She initially presented with back pain, dizziness, and progressive lower limb weakness, which evolved into complete immobility. Neurological examination revealed ascending weakness, bilateral lower motor neuron facial nerve palsy, bulbar symptoms, urinary retention, and areflexia. Cerebrospinal fluid analysis demonstrated albuminocytologic dissociation. Nerve conduction studies showed features consistent with acute inflammatory demyelinating polyradiculoneuropathy (AIDP), including prolonged distal motor latencies, reduced conduction velocities, and absent F-waves. The patient was treated with a five-day course of intravenous immunoglobulin (IVIg), along with supportive care including thromboprophylaxis, pain management, and close monitoring of respiratory and autonomic function. This case highlights the importance of considering GBS in postpartum patients with evolving neuromuscular symptoms and reinforces the need for timely diagnosis and intervention to improve outcomes.

## Introduction

Guillain-Barré syndrome (GBS) is a rare autoimmune disorder with an annual incidence of one to two per 100,000 people [1], in which the body’s immune system attacks the peripheral nervous system, leading to muscle weakness, sensory disturbances, and, in severe cases, paralysis. The condition is often triggered by infections, but it can also be induced by physical trauma, vaccinations, or other immune responses. Up to 30% of cases show no identifiable trigger [2]. GBS typically presents as acute ascending paralysis, often starting with weakness in the legs [3]. Early diagnosis and prompt treatment are critical to improving patient outcomes.

The postpartum period has been identified as a time when women may be at an increased risk of developing GBS, especially within the first 30-90 days following delivery [4]. The postpartum immune rebound, marked by pro-inflammatory cytokine restoration, has been linked with autoimmune flares, including GBS. The return of the immune system to normal levels is crucial for preventing flares of autoimmune conditions [5]. This report discusses the case of a patient who developed GBS three months after delivery, with no identifiable infectious trigger, adding to the growing literature showing postpartum onset in the absence of infection but following immunity-affecting obstetric events [6].

## Case presentation

A young female presented to the emergency department with complaints of back pain and bilateral lower limb weakness, which she described as a “jelly-like” feeling in her legs. The patient had previously consulted her general practitioner a week earlier for symptoms of dizziness, back pain, and ataxia. She was prescribed prochlorperazine and sent home. However, her symptoms worsened, with increasing back pain and bilateral leg weakness, prompting her to seek further medical attention in the emergency department.

The patient’s medical history was unremarkable, except for a vaginal delivery three months earlier, which had been complicated by a retained placenta that was manually removed. She was previously independent and mobile, living with her family. The patient was a non-smoker and consumed alcohol occasionally. Her maternal uncle had multiple sclerosis, but there was no other significant family history.

To rule out cauda equina syndrome, an MRI of the lumbar spine was performed, but the results were unremarkable. The patient was discharged; however, she later returned with worsening bilateral leg weakness and was referred to the medicine department with a suspected diagnosis of GBS. Upon this second presentation to the emergency department, a CT scan of the brain was done, along with blood tests, including a full blood count and inflammatory markers, which showed no significant findings. A lumbar puncture revealed elevated protein levels and a normal white blood cell count, suggesting albuminocytologic dissociation. The results are outlined in Table 1.

**Table 1:** Lumbar puncture findings LP: lumbar puncture; CSF: cerebrospinal fluid; WBC: white blood cell; RBC: red blood cell

Test	Patient Value	Reference Range
LP Protein	1.30 g/L	<0.5 g/L
CSF IgG	0.148	0.001-0.042
CSF Albumin	0.75	0.10-0.40
CSF Wbc	1	0-5 cells/microlitre
CSF Rbc	10	0 cells/microlitre
CSF Gram stain	Negative	

On physical examination, the patient was unsteady on her feet and exhibited ascending weakness. The onset of the patient’s symptoms occurred in the 10th week following delivery, with a rapid progression thereafter, reaching maximal severity within an eight-day period. She was completely unable to mobilize without assistance, struggled with writing and using the phone, experienced changes in speech, and developed facial asymmetry. She also developed urinary retention. Her extraocular movements remained normal. Neurological exam showed bilateral lower motor neuron facial nerve (cranial nerve VII) palsy, asymmetric but intelligible bulbar speech, and strong tongue function. Motor exam revealed subtle upper limb flaccidity (hand grip 5/5, elbow flexion 4/5, shoulder 3/5), reduced hip flexion (2/5), and 4/5 strength in hip extension, knee extension/flexion, and ankle movements. Reflexes were absent at the knees and ankles, with intact sensation.

**Figure 1:**
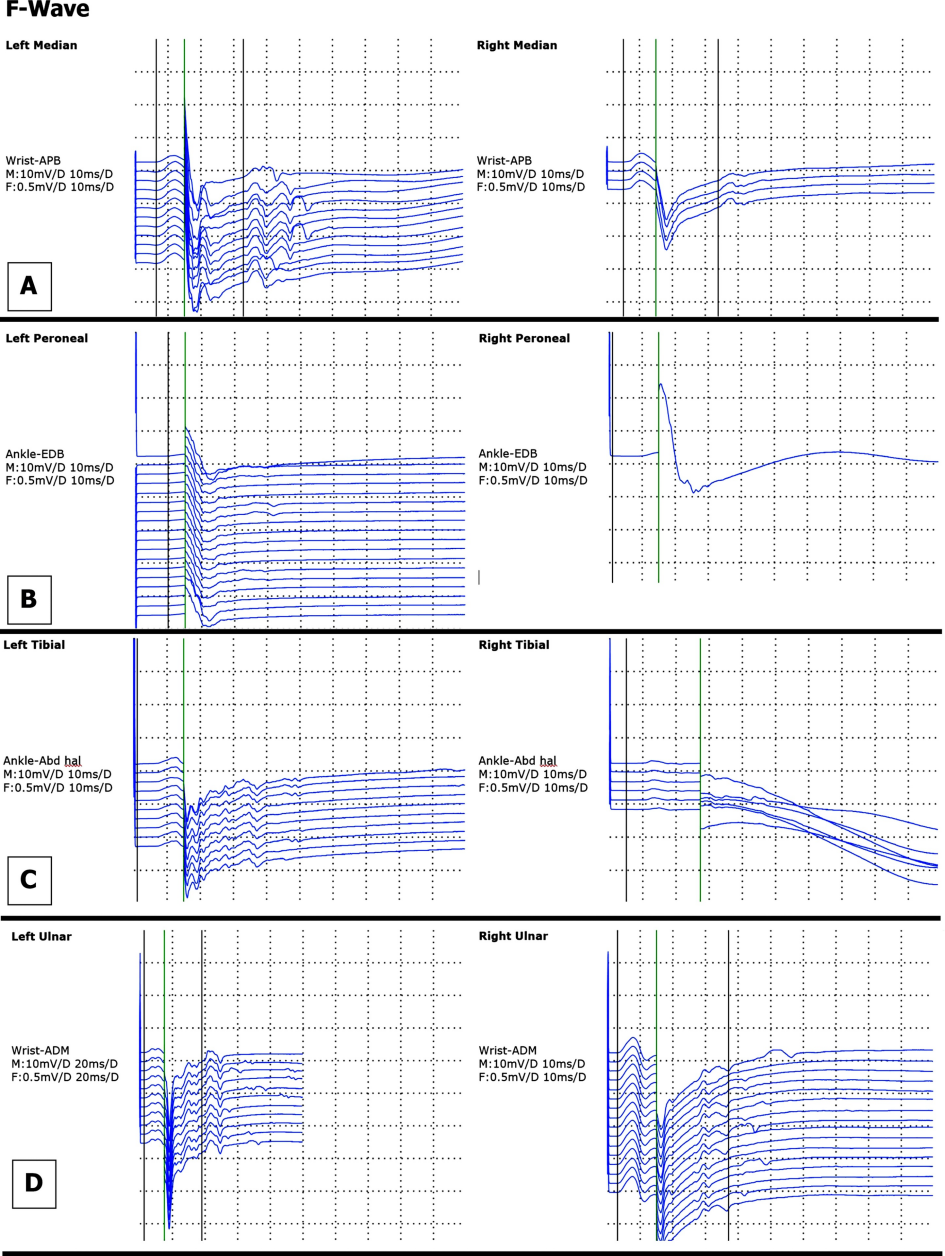
F-waves of nerve conduction studies F-Wave Curves: (A) Right and Left Median Nerve; (B) Right and Left Peroneal Nerve; (C) Right and Left Tibial Nerve; (D) Right and Left Ulnar Nerve

**Figure 2:**
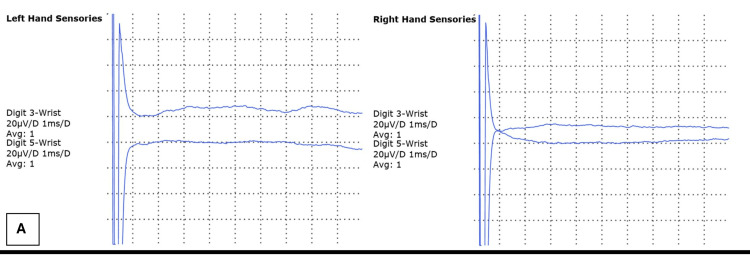
Sensory nerve conduction curves: right vs. left hand

**Figure 3:**
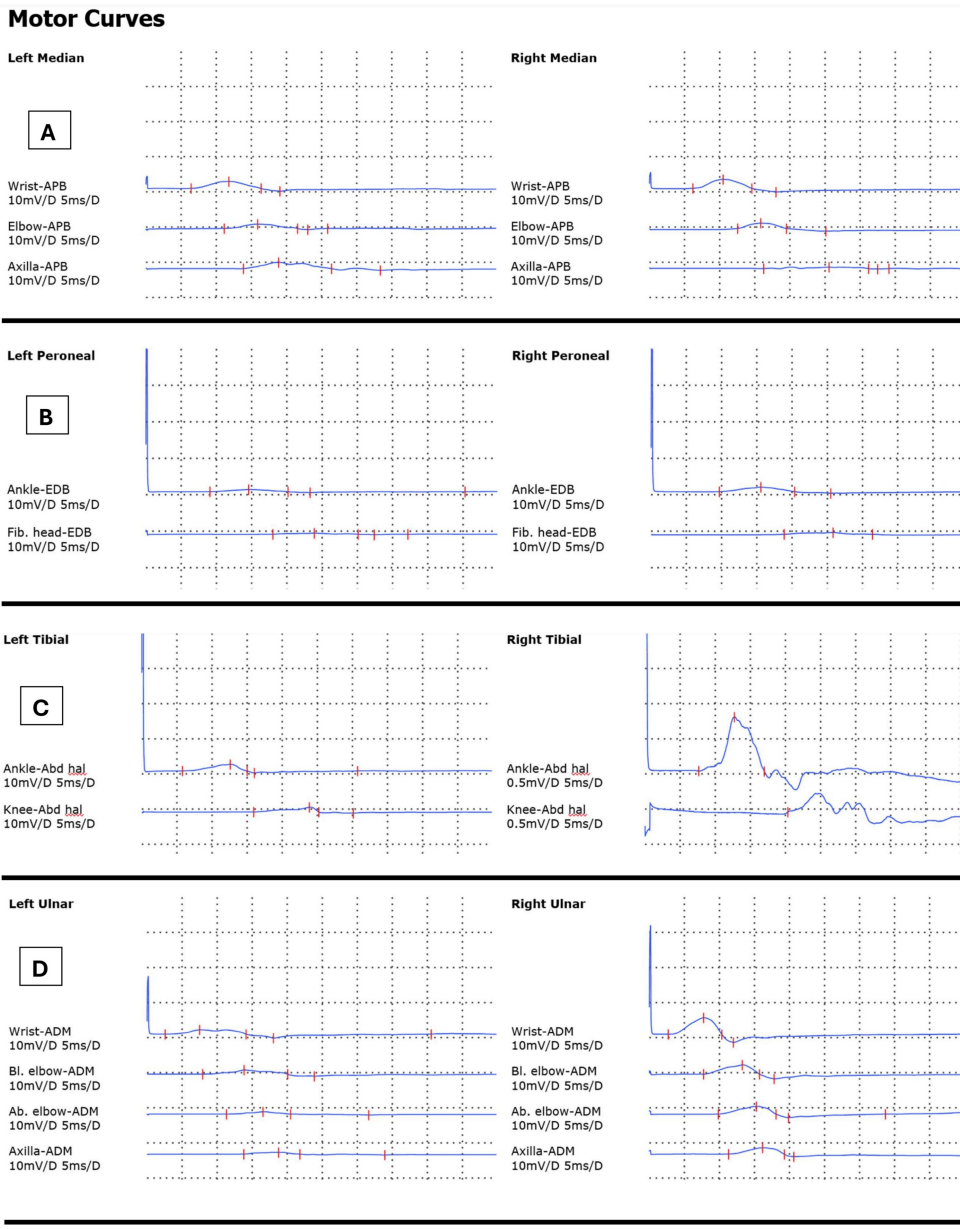
Motor nerve conduction studies (A) Right vs. Left Median Nerves; (B) Right vs.Left Peroneal Nerves; (C) Right vs.Left Tibial Nerves; (D) Right vs.Left Ulnar Nerves

Nerve conduction studies (NCS) demonstrated a bilateral sensory and motor neuropathy with both axonal and demyelinating features. Specifically, there was prolonged distal motor latency, slowed conduction velocity in multiple nerves, reduced amplitudes, absent F-waves in more than one nerve (Figure 1), and evidence of proximal conduction abnormalities. Sensory nerve conduction graphs are presented in Figure 2, and motor nerve conduction findings are illustrated in Figure 3. Notably, sural responses were preserved.

These findings meet the electrodiagnostic criteria for acute inflammatory demyelinating polyneuropathy (AIDP), with possible secondary axonal involvement. Needle EMG demonstrated reduced activity in the right leg muscles (Table 2), further supporting the diagnosis.

**Table 2:** Summary of electromyography findings PSW: positive sharp waves; CRD: complex repetitive discharges; IP: interference pattern

Muscle	Interpretation	Insertion	Spontaneous Activity					Voluntary Activity						Notes
		Activity	Fibrillation potential	PSW	Fasciculation	Myotonia	CRD	Amplitude	Duration	Polyphasia	Stability	IP	Amplitude	
Right Gastroc caput med	Normal	Normal	None	None	None	None	None	Normal	Normal	None	Normal	Discrete	Normal	Reduced activity
Right Tibialis anterior	Normal	Normal	None	None	None	None	None	Normal	Normal	None	Normal	Discrete	Normal	Normal recruitment
Right Vastus med	Normal	Normal	None	None	None	None	None	Normal	Normal	None	Normal	Discrete	Normal	Within normal limits

After the diagnosis, the patient was started on intravenous immunoglobulin (IVIg) therapy at a dose of 0.4 g/kg/day for five consecutive days. Prophylactic low-molecular-weight heparin (LMWH) was administered to reduce the risk of venous thromboembolism. Intravenous fluids were provided at a rate of 2 liters over 24 hours to maintain adequate hydration. Gabapentin was prescribed at a dose of 200 mg three times daily for the management of radicular pain and paresthesia, given its favorable side effect profile and proven efficacy in treating neuropathic pain. Laxatives were given to address constipation.

Daily electrocardiogram (ECG) monitoring was performed to assess for potential autonomic or cardiac complications. Due to dysarthria, a speech and language therapy (SALT) assessment was requested, and the patient was kept nil by mouth (NPO) pending evaluation. Additionally, respiratory function was closely monitored through serial assessments of single breath count every two hours. The GBS Disability Scale was used to track neurological recovery. On admission, the patient was scored at Grade 4, indicating she was bedridden or chairbound. Over time, her condition gradually improved, reaching Grade 2 at four months, where she was able to walk with assistance but not independently.

## Discussion

GBS remains a complex and unpredictable disorder, with its precise triggers sometimes difficult to pinpoint. In this case, the absence of a clear infectious or immunological precursor highlights the variability in GBS presentations and underscores the need to consider less common contributing factors. This case adds to the growing body of evidence suggesting that pregnancy-related immune modulation can be a potential risk factor even months after delivery.

According to the case series by Mirawati et al. (2024) , a significant proportion of postpartum GBS cases occur within 6-12 weeks of delivery [7]. Iliadi-Tulbure et al. (2024) highlighted that IVIg was effective in reversing limb and cranial deficit [8]. The manual removal of the retained placenta may have triggered a stress-induced immune response, potentially contributing to the development of GBS in this patient [9]. Multiple postpartum shifts ranging from IL-6 surges to tissue breakdown can induce autoimmune responses, particularly after obstetric complications [10].

Atypical variants of GBS in pregnant or postpartum women have been documented. Wang et al. (2023) [11] and Krief et al. (2023) [12] emphasized that when initial imaging and laboratory results are unremarkable, physicians should focus on clinical progression and cerebrospinal fluid analysis to ensure timely diagnosis. Our patient met the classic criteria of albuminocytologic dissociation and was promptly started on immunoglobulin therapy.

The rapid progression of symptoms, including the development of cranial nerve involvement and autonomic dysfunction, is characteristic of severe GBS, particularly the AIDP subtype. In this case, symptoms began with mild weakness and back pain on day one and progressed to peak severity by day eight, marked by paralysis, bilateral facial nerve palsy, and urinary retention, consistent with the typical progression diagnostic of GBS. Electrophysiologic studies confirmed the AIDP subtype, which correlates with the clinical severity observed. Timely administration of IVIg was crucial in improving patient outcomes.

## Conclusions

This case highlights the critical importance of early recognition and treatment of GBS, especially in the presence of cranial nerve involvement and autonomic dysfunction. The postpartum period is a significant risk window, with GBS potentially developing up to three months after delivery, particularly following complications like manual placenta removal. Early administration of IVIg remains the cornerstone of effective treatment and can improve patient outcomes. This report adds to the growing body of literature on postpartum GBS without preceding infections and reinforces the need to consider GBS in the differential diagnosis of back pain, ataxia, and limb weakness.
